# Fulvic acid attenuates homocysteine-induced cyclooxygenase-2 expression in human monocytes

**DOI:** 10.1186/s12906-015-0583-x

**Published:** 2015-03-13

**Authors:** Shao-Ju Chien, Te-Chuan Chen, Hsing-Chun Kuo, Cheng-Nan Chen, Shun-Fu Chang

**Affiliations:** Division of Pediatric Cardiology, Department of Pediatrics, Kaohsiung Chang Gung Memorial Hospital, Chang Gung University College of Medicine, Kaohsiung, Taiwan; Division of Nephrology, Kaohsiung Chang Gung Memorial Hospital and Chang Gung University College of Medicine, Kaohsiung, Taiwan; Institute of Nursing and Department of Nursing, Chang Gung University of Science and Technology, Taoyuan, Taiwan; Chronic Diseases and Health Promotion Research Center, Chang Gung University of Science and Technology, Taoyuan, Taiwan; Research Center for Industry of Human Ecology, Chang Gung University of Science and Technology, Taoyuan, Taiwan; Department of Biochemical Science and Technology, National Chiayi University, Chiayi, 600 Taiwan; Biophotonics and Molecular Imaging Research Center, National Yang Ming University, No. 155, Sec. 2, Linong St. Beitou District, Taipei, Taiwan

**Keywords:** Anti-inflammation, Cyclooxygenase-2, Fulvic acid, Homocysteine, Monocytes

## Abstract

**Background:**

Homocysteine and pro-inflammatory mediators such as cyclooxygenase-2 (COX-2) have been linked to vascular dysfunction and risks of cardiovascular diseases. Fulvic acid (FA), a class of compounds of humic substances, possesses various pharmacological properties. However, the effect of FA on inflammatory responses of the monocytes remains unclear. We investigated the regulatory effect of FA on homocysteine-induced COX-2 expression in human monocytes.

**Methods:**

Peripheral blood monocytes and U937 cells were used for all experiments. Real-time PCR and ELISA assay were used to analyze the COX-2 mRNA expression and PGE2 secretion, respectively. Specific inhibitors were used to investigate the mechanism of homocysteine-mediating COX-2 mRNA expression and PGE2 secretion. Luciferase assay, transcription factor ELISA, and chromatin immunoprecipitation were used to determine the role of nuclear factor-κB in FA-mediated inhibition of homocysteine effect on monocytes.

**Results:**

The results show that pretreating monocytes with FA inhibited the homocysteine-induced COX-2 expression in a dose-dependent manner. Stimulation of U937 monocytes with homocysteine induced rapid increases in the phosphorylation of ERK and JNK; the inhibitor for ERK and JNK attenuated the homocysteine-induced nuclear factor-κB activation and COX-2 expression. Transcription factor ELISA and chromatin immunoprecipitation assays showed that FA blocked the homocysteine-induced increases in the binding activity and in vivo promoter binding of nuclear factor-κB in monocytes.

**Conclusions:**

Our findings provide a molecular mechanism by which FA inhibits homocysteine-induced COX-2 expression in monocytes, and a basis for using FA in pharmaceutical therapy against inflammation.

## Background

Fulvic acid (FA), a class of compounds of humic substances, is a mixture of polyphenolic acid compounds formed through the degradation of organic substances such as dead plants, microbes and animals by chemical and biological processes [1]. FA has been reported recently to have nutraceutical properties and physiological action on the human body. It is one of the most interesting naturally occurring phytochemicals with its neuroprotective effect [2,3]. Antimicrobial and anti-inflammatory properties of FA have also been reported [4,5]. In addition, the FA extracted from peat had an antioxidant activity and an inhibitory effect on chemical mediator release in basophils [6]. These results imply that the FA may possess a predominant role in their biological activity. Although there are a number of studies on the effect of FA on cellular and biological functions, the detailed mechanisms underlying the regulatory effect of FA remain unclear.

The formation of atherosclerotic lesions is regarded as a process of chronic inflammatory responses [7]. Several risk factors are known to be involved in promoting atherosclerosis, including smoking, diabetes mellitus, hyperlipidemia, hypertension, and hyperhomocysteinemia. Abnormal elevation of homocysteine levels in the blood have been reported in patients with hyperhomocysteinemia [8]. Severe hyperhomocysteinemia (plasma levels of homocysteine greater than 100 mmol/L) is found in patients with extremely premature atherosclerosis and early occlusive vascular disease [9]. Although hyperhomocysteinemia has been considered an independent risk factor for atherosclerosis, the mechanism of causing vascular damage by homocysteine is not yet understood. Endothelial dysfunction and activation is one of the key events in vascular pathology associated with homocysteine [10]. In addition, oxidative stress, inflammation, and smooth muscle cell proliferation, are also involved in this process.

Monocytes are the primary inflammatory cell type that infiltrates early atherosclerotic plaques. The release of pro-inflammatory mediators by monocyte-derived macrophages may play a crucial role in atherosclerotic inflammatory responses [11]. Cyclooxygenase-2 (COX-2) is a key enzyme for the synthesis of eicosanoids. COX-2 is considerably expressed in vascular tissues due to the stimulation of pro-inflammatory factors, such as cytokines, mitogens and lipopolysaccharide [12]. It is evident that COX-2 in activated monocytes is of particular relevance in inflammation and atherosclerosis [13]. The activation of macrophages has been previously correlated with the induction of COX-2. Macrophages expressing COX-2 are known to produce prostaglandins that have pro-inflammatory effects, including activating chemotaxis, increasing vascular permeability, and promoting cell proliferation [12]. Since atherosclerosis is a multifactorial disease involving a complex array of contributing factors including monocyte functions and hyperhomocysteinemia, it is necessary to investigate the effect of homocysteine on COX-2 gene expression in human monocytes. In the present study, we investigated the roles of FA in modulating homocysteine-induced COX-2 expression in primary human blood monocytes and human monocytic U937 cells, and also the molecular mechanisms underlying the regulatory effects.

## Methods

### Materials

All culture materials were purchased from Gibco (Grand Island, NY, USA). FA was supplied as a 20% solution by Esther Material Technology Co., Ltd., Kaohsiung, Taiwan. PD98059 (ERK inhibitor), SP600125 (JNK inhibitor), and SB203580 (p38 inhibitor) were purchased from Calbiochem (La Jolla, CA). Mouse monoclonal antibodies (mABs) against ERK1/2, JNK1/2, phospho-ERK1/2, phospho-JNK1/2, and NF-κB p65 were purchased from Santa Cruz Biotechnology (Santa Cruz, CA). Pyrrolidine dithiocarbamate (PDTC), SN50, and other chemicals of reagent grade were obtained from Sigma (St. Louis, MO).

### Cell culture

Human monocytes from the buffy coat (Taiwan Blood Center, TBSF, Taiwan) were isolated as previously described [14]. Peripheral blood mononuclear cells (PBMCs) were isolated by Histopaque 1077 density-gradient centrifugation. Monocytes were purified from PBMCs by negative selection using the magnetic-activated cell sorting (MACS) monocyte isolation kit (Miltenyi Biotech, Auburn, CA). The human monocytic cell line U937 was obtained from the Bioresources Collection and Research Center (BCRC) of the Food Industry Research and Development Institute (Hsinchu, Taiwan). Cells were maintained in RPMI-1640 medium supplemented with 10% FBS.

### Real-time quantitative PCR

Real-time PCR was performed, and products were detected using an ABI Prism 7900HT with the FastStart DNA SYBR Green I kit (Roche Diagnostics GMbH, Mannheim, Germany). The designed primers in this study were COX-2 forward primer, 5′-CTGAA AGATG GACGC TCAAT-3′; COX-2 reverse primer, 5′-CGTTT CAGAA GCCAG AAGAG-3′; 18S rRNA forward primer, 5′-CGGCG ACGAC CCATT CGAAC-3′, 18S rRNA reverse primer, 5′-GAATC GAACC CTGAT TCCCC GTC-3′. Quantification was performed using the 2^−ΔΔCt^ method [15]. All samples were measured in duplicate. The average value of both duplicates was used as the quantitative value.

### PGE_2_ assay

The levels of PGE_2_ in the conditioned media were determined by using the PGE_2_ ELISA assay kit (R & D Systems) according to the manufacturer’s instructions [16].

### Western blot analysis

Samples were lysed with a buffer containing 1% NP-40, 0.5% sodium deoxycholate, 0.1% SDS, and a protease inhibitor mixture (PMSF, aprotinin, and sodium orthovanadate). The protein concentration was determined using the Bio-Rad protein assay kit (Bio-Rad, Hercules, CA). Equal amounts of total proteins were separated by SDS-polyacrylamide gel electrophoresis (PAGE) (10% running, 4% stacking), transferred onto a nitrocellulose membrane, and analyzed using the designated antibodies and the Western-Light chemiluminescent detection system (Bio-Rad).

### Luciferase assays

Human COX-2 promoter constructs containing −918/+49 of COX-2 5′-flanking DNA linked to the firefly luciferase reporter gene of plasmid pGL4 (Promega, Madison, WI) were used as previously reported.^16^ DNA plasmids at a concentration of 1 mg/ml were transfected into DLD-1 cells by Lipofectamine (Gibco). The pSV-β-galactosidase plasmid was cotransfected to normalize the transfection efficiency. Values obtained were normalized to the levels of β-galactosidase in the cell lysates. β-galactosidase activities were determined with an assay kit and exhibited <20% variation between samples.

### Transcription factor assays (TF ELISA assays)

Nuclear extracts of cells were prepared using nuclear protein extract kits (Panomics, Redwood City, CA). Equal amounts of nuclear proteins were employed for quantitative measurements of NF-κB p65 activation using commercially available ELISA kits (Panomics).

### Chromatin immunoprecipitation (ChIP)

ChIP assays were performed following a published protocol [17]. Briefly, chromatins were sheared by sonication (3 times, 10 sec on, 60 sec off). Precleared extracts were immunoprecipitated with rabbit anti-p65 and c-jun antibodies or rabbit IgG at 4°C overnight. DNA was isolated from precipitated complexes and analyzed by PCR with the following primers that amplify the part of the human COX-2 promoters that contain the NF-κB binding sites: 5′-GCCCT CCCCC GGTAT CCCAT C-3′ and 5′-AAAAA ATTGC GTAAG CCCGG T-3′. An aliquot of total input nuclear extract was used as the loading control.

### Statistical analysis

The results are expressed as the mean ± standard error of the mean (SEM). Statistical analysis was determined using an independent Student *t*-test for two groups of data and analysis of variance (ANOVA) followed by the Scheffe’s test for multiple comparisons. *P* values less than 0.05 were considered significant.

## Results

### Cytotoxic effect of FA on human monocytes

**Figure 1:**
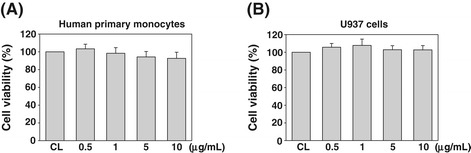
**The effect of fulvic acid (FA) on cell viability of human primary and U937 monocytes.** Human primary monocytes **(A)** and U937 cells **(B)** were cultured with the indicated concentrations of FA and incubated at 37°C in a 96-well plate for 24 h. Cell viability was evaluated as described in the “Methods Section”, and is expressed as a percentage of the control cells (CL). Values are expressed as the mean ± standard error of the mean (SEM) of three individual experiments.

To examine the effect of FA on the viability of monocytes, human primary monocytes and U937 cells were treated with FA at a concentration of 0.5, 1, 5, or 10 μg/mL for 24 h, and the MTT assay was performed. As shown in Figure 1, there was no significant difference on the cell viability between FA-treated and untreated human primary monocytes (Figure 1A) and U937 cells (Figure 1B). These results indicate that the FA used in the present study has no cytotoxic effect on monocytes.

### FA inhibits homocysteine-induced COX-2 expression in monocytes

**Figure 2:**
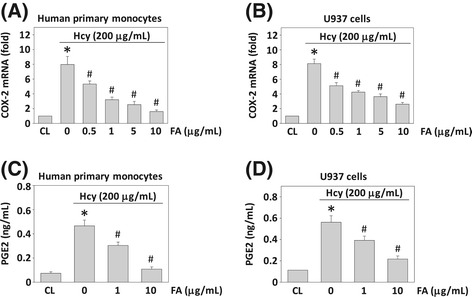
**The effect of fulvic acid (FA) on homocysteine-induced COX-2 mRNA expression and PGE**_**2**_ **secretion in human monocytes.** Human primary monocytes and U937 cells were pre-treated with FA (0–10 μg/mL) for 4 h, and then stimulated with homocysteine (200 μg/mL) for 4 h **(A, B)** and 8 h **(C, D)**. Monocytes that were not stimulated with homocysteine were used as controls (CL). **(A, B)** RNA samples were isolated and subjected to real-time PCR analysis. Data are presented as fold changes in fluorescent density from CL monocytes normalized to 18S rRNA level of three individual experiments. **(C, D)** The PGE_2_ secretion in conditioned media was determined by ELISA analyses. Data are shown as mean ± standard error of the mean (SEM) of three individual experiments. *P < 0.05 versus CL monocytes. ^#^ *P* < 0.05 versus homocysteine-stimulated cells without pretreatment of FA.

To test the effects of FA on homocysteine-induced COX-2 expression in monocytes, human primary monocytes and U937 cells were pretreated with FA at concentrations of 0.5, 1, 5, and 10 μg/mL for 4 h, and then stimulated with homocysteine (200 μM) for 4 h in the presence of FA. The results from real-time PCR analysis showed that homocysteine induced a significant increase in the monocytic COX-2 mRNA expression, as compared with the unstimulated cells (Figure 2A for human primary monocytes, Figure 2B for U937 cells). This increase in COX-2 mRNA expression was significantly inhibited by pretreating cells with FA (Figure 2A and B) and the inhibitory effect of FA is in a dose dependent manner. ELISA assays for PGE_2_ secretion in conditioned medium showed that the stimulation of monocytes with homocysteine resulted in the increase in PGE_2_ secretion from the monocytes, as compared with the unstimulated cells (Figure 2C for human primary monocytes, Figure 2D for U937 cells). Pretreating monocytes with FA at a concentration of 1 or 10 μg/mL reduced the homocysteine-induced PGE_2_ secretion. This result shows that the effect of FA on homocysteine-induced COX-2 gene expression is accompanied by the corresponding changes of the PGE_2_ release from monocytes.

### FA mediates homocysteine-induced COX-2 gene expression at the transcriptional level

**Figure 3:**
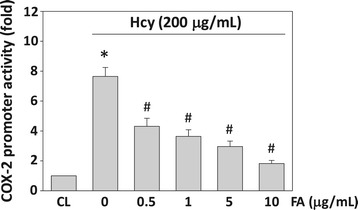
**Fulvic acid (FA) modulation of homocysteine-induced COX-2 expression is a transcriptional event.** U937 cells were transfected with COX-2 p918-Luc promoter plasmid. Cells transfected with p918-Luc that were not stimulated with homocysteine were used as controls (CL). COX-2 promoter activity was measured as fold changes using luciferase assay normalized to β-galactosidase activity from at least three individual experiments. *P < 0.05 versus CL monocytes. ^#^ *P* < 0.05 versus homocysteine-stimulated cells without pretreatment of FA.

To further determine whether the FA modulations of homocysteine-induced COX-2 expression are transcriptional events, U937 cells were transiently transfected with the COX-2 promoter construct containing the promoter region of COX-2 and the reporter gene luciferase (p918-Luc), pretreated with FA for 4 h, and then treated with homocysteine for 4 h. U937 cells treated with homocysteine for 4 h significantly increased the COX-2 promoter activity by approximately 7.65-fold compared with the unstimulated cells (Figure 3). Pretreatment of the cells with FA (0.5-10 μg/mL) for 4 h significantly attenuated this homocysteine-induced COX-2 promoter activity. These results suggest that FA-mediated COX-2 induction by homocysteine is regulated at the transcriptional level.

### FA-mediated inhibition of homocysteine-induced COX-2 expression is dependent on MAPK

**Figure 4:**
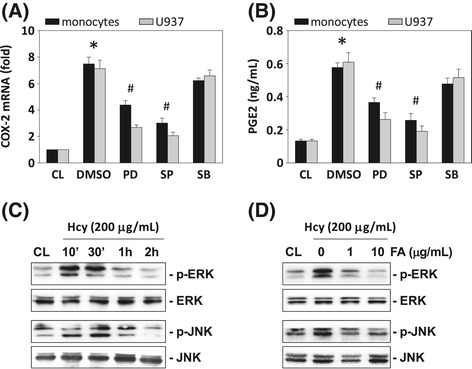
**The effect of fulvic acid (FA) on homocysteine-induced phosphorylation of MAPKs signal pathways in human monocyte.** Human primary monocytes and U937 cells were kept as CL or stimulated with 200 μg/mL homocysteine for 4 h **(A)** or 8 h **(B)**. Before being kept as CL or stimulated with homocysteine, cells were pretreated with PD98059 (PD), SP600125 (SP), or SB203580 (SB) individually for 1 h. **(A)** All bar graphs represent folds of CL monocytes and normalized to 18S rRNA. **(B)** PGE_2_ secretion was determined by ELISA assay. The results are shown as mean ± SEM of three individual experiments. *P < 0.05 versus CL. ^#^ *P* < 0.05 versus vehicle control (DMSO) with homocysteine stimulation. **(C)** U937 cells were kept as CL or stimulated with homocysteine for times indicated. **(D)** U937 monocytes were pre-treated with FA (0–10 μg/mL) for 4 h, and then stimulated with homocysteine for 0.5 h. The phosphorylation of ERK and JNK was determined by Western blotting. The results shown are representative of three independent experiments with similar results.

To determine whether the homocysteine-induced COX-2 expression is mediated through the MAPK-dependent pathways, human primary monocytes and U937 cells were incubated with a specific inhibitor for ERK (PD98059, 30 mM), JNK (SP600125, 20 mM), or p38 (SB203580, 10 mM) for 1 h before and during stimulation with 200 μM homocysteine. The homocysteine-induced COX-2 mRNA expression (Figure 4A) and PGE_2_ secretion (Figure 4B) were significantly inhibited by PD98059 and SP600125, but not by SB203580. Moreover, the phosphorylation of ERK and JNK in U937 cells also increased rapidly after homocysteine stimulation, reaching maximal levels at 10–30 min (Figure 4C). After these transient increases, phosphorylation decreased to nearly basal levels. U937 cells treated with FA (1 and 10 μg/mL) for 4 h before the addition of homocysteine significantly inhibited the homocysteine-induced ERK and JNK phosphorylation (Figure 4D). FA itself had no effect on the basal levels of ERK, JNK, and p38 MAPK phosphorylation in the control cells (data not shown). These results suggest that the inhibitory effect of FA on homocysteine-induced COX-2 expression was attributable to their inhibition in homocysteine-induced ERK and JNK activation in monocytes.

### FA-mediated inhibition of homocysteine-induced COX-2 expression is dependent on NF-κB

**Figure 5:**
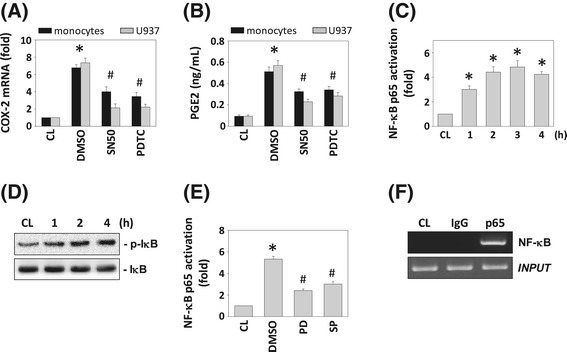
**The roles of NF-κB in homocysteine-induced COX-2 mRNA expression and PGE**_**2**_ **secretion. (A)** COX-2 mRNA and **(B)** PGE_2_ expression were determined in human primary monocytes and U937 cells pretreated with NF-κB inhibitors PDTC and SN50, and then stimulated with 200 μg/mL homocysteine for 2 h. All bar graphs represent folds of CL monocytes, mean ± SEM of three individual experiments. *P < 0.05 versus CL. ^#^ *P* < 0.05 versus DMSO-treated cells with homocysteine stimulation. **(C)** and **(E)** NF-κB activation was determined by TF ELISA assays in U937 cells treated with homocysteine only **(C)** and both homocysteine and PD98059/SP600125 **(E)**. *P < 0.05 versus CL. **(D)** The phosphorylation of IκB was determined by Western blotting. **(F)** ChIP assays were performed for NF-κB using p65 antibody. The results shown are representative of three independent experiments with similar results.

**Figure 6:**
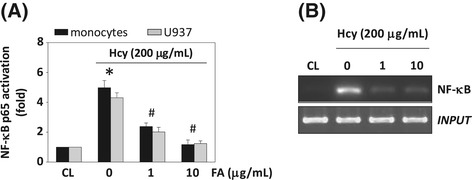
**The effect of fulvic acid (FA) on homocysteine-induced NF-κB activation in U937 monocyte.** U937 cells were kept as CL or stimulated with 200 μg/mL homocysteine for 4 h. Before being kept as CL or stimulated with homocysteine, cells were pretreated FA (0–10 μg/mL) for 4 h. **(A)** NF-κB activation was determined by TF ELISA assays in U937 cells. *P < 0.05 versus CL. **(B)** ChIP assays were performed for NF-κB using p65 antibody. The results shown are representative of three independent experiments with similar results.

The COX-2 gene contains NF-κB binding elements in its promoter region [16]. To investigate whether NF-κB is involved in FA-mediated inhibition of homocysteine-induced COX-2 expression in monocytes, we examined the effects of NF-κB inhibitors on the homocysteine-induced COX-2 expression in human primary monocytes and U937 cells by using the NF-κB inhibitors SN50 and PDTC. Human primary monocytes and U937 cells were incubated with specific inhibitors of NF-κB (SN50 and PDTC, 50 mM) for 1 h, followed by treatment with homocysteine for 4 h. The homocysteine-induced COX-2 mRNA expression (Figure 5A) and PGE_2_ secretion (Figure 5B) were significantly reduced by inhibition with PDTC and SN50, indicating that NF-κB is involved in the regulation of COX-2 gene induction. To investigate whether NF-κB binds the COX-2 promoter region in U937 cells, we determined the NF-κB activation by using TF ELISA assay and IκB phosphorylation analysis. These results showed that the treatment of U937 cells with homocysteine caused NF-κB p65 activity (Figure 5C) and IκB phosphorylation (Figure 5D) to increase at 1 h and remain elevated for at least 4 h. Moreover, pretreating cells with ERK- or JNK-specific inhibitor significantly attenuated homocysteine-increased NF-κB p65 activity (Figure 5E). To confirm these results, ChIP analysis was performed. Immunoprecipitated chromosomal DNA with anti-NF-κB p65 antibody was subjected to PCR using primers designed to amplify the COX-2 promoter region harboring the NF-κB binding sites. NF-κB p65 indeed bound to the COX-2 promoter region containing the NF-κB sites (Figure 5F). To determine whether FA would mediate the NF-κB p65-DNA binding activity in the nucleus of monocytes in response to homocysteine, we performed TF ELISA assays. As shown in Figure 6A, pretreatment of U937 cells with FA (1 and 10 μg/mL) for 4 h decreased the homocysteine-induced NF-κB-DNA binding activity. To further assess the *in vivo* regulation of the binding of NF-κB to the promoter regions of the COX-2 gene in monocytes stimulated with homocysteine in the presence of FA, we performed ChIP assays in U937 cells by using an antibody against p65. The homocysteine-induced *in vivo* NF-κB p65 binding to the COX-2 promoter was significantly inhibited by pretreating the cells with FA for 4 h (Figure 6B).

## Discussion

**Figure 7:**
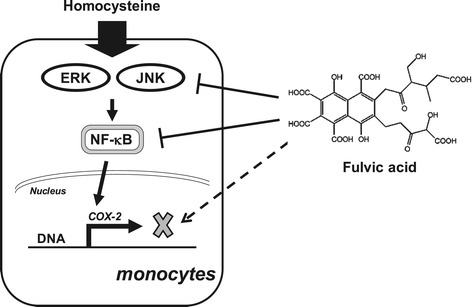
**Schematic diagram showing the FA structure and the antagonistic effects of FA on homocysteine-induced inflammatory COX2 expression in human monocytes.**

Homocysteine is thought to be an independent risk factor for atherosclerosis in human beings. Multifactorial mechanisms such as oxidative stress and inflammation have been found to play a role in hyperhomocysteinemia-induced atherogenesis [18,19]. In addition, previous study suggested that elevated levels of homocysteine may not directly induce atherogenesis, but may instead accelerate atherosclerotic lesion development in combination with other cardiovascular risk factors [20]. The production of pro-inflammatory mediators such as COX-2 in monocytes plays an important role in atherogenesis [21]. However, the mechanism by which homocysteine regulates COX-2 gene expression of monocytes remains unclear. Fulvic acid is one of the most interesting phytocomplex molecules and is reported to have several nutraceutical properties with potential anti-oxidant and anti-inflammatory activities [22]. Our present study demonstrates for the first time that FA can exert inhibitory effects on homocysteine-induced COX-2 expression in monocytes, thereby possibly serving anti-inflammatory and atheroprotective functions. This inhibitory effect of FA on homocysteine-induced COX-2 expression was mediated by the ERK/JNK and NF-κB signaling pathways based on several lines of evidence (Figure 7). First, pretreatment of human primary monocytes and U937 cells with FA inhibited the homocysteine-induced COX-2 expression in a dose-dependent manner. Second, FA inhibited the homocysteine-induced COX-2 promoter activity. This suggests that the FA-mediated inhibition in homocysteine-induced COX-2 expression was regulated at the transcriptional level. Third, treating cells with homocysteine induced rapid increases in their ERK and JNK phosphorylation; the inhibitors for ERK and JNK attenuated the homocysteine-induced COX-2 expression. Pretreating U937 cells with FA inhibited the homocysteine-induced ERK and JNK activation, suggesting that the ERK and JNK pathways were involved in the inhibitory effect of FA on homocysteine-induced COX-2 expression. Finally, homocysteine induced the NF-κB p65-DNA binding activity and in vivo promoter binding of NF-κB in U937 cells. These increases in the NF-κB activation and NF-κB-promoter binding could be inhibited by pretreating the human primary monocytes and/or U937 cells with FA.

Monocytes are one of the main cell types that express COX-2 [23]. Transendothelial migration of monocytes into the vessel wall is the initial step in the formation of atherosclerotic lesions. Previous study has indicated that COX-2 up-regulation was observed in peripheral blood monocytes from patients with acute myocardial infarction, suggesting that an acute inflammatory response to acute myocardial infarction is correlated with COX-2 activation in peripheral blood monocytes [24]. In addition, the presence of COX-2 has also been reported in the shoulder region of atherosclerotic plaques, mainly colocalizing with macrophages and matrix metalloproteinases, which leads to vascular remodeling and atherothrombotic syndromes [25]. The results of this study demonstrate that homocysteine induces both COX-2 gene expression and PGE_2_ secretion in human monocytes. It has been reported that homocysteine induced COX-2 expression in murine macrophages by ROS generated via NMDA receptor-mediated calcium-signaling pathways [26]. In the present study, we found that homocysteine induced rapid increases in the phosphorylation of ERK and JNK in U937 cells. Furthermore, a specific inhibitor for ERK and JNK inhibited the homocysteine-induced COX-2 expression. These results indicate that the activation of ERK and JNK is critical for the homocysteine-induction of COX-2. Our present study further demonstrated that FA has an inhibitory effect on the homocysteine-induced ERK and JNK phosphorylation, and COX-2 expression.

The transcription factor NF-κB plays a critical role in regulating inducible gene expression in inflammatory responses. NF-κB in the promoter regions of the COX-2 gene have been shown to be essential for the responsiveness of this gene to stimuli [27]. NF-κB dimers are retained in an inactive form in the cytosol through their interaction with IκB proteins. The interaction of pro-inflammatory factors on cells induces phosphorylation and degradation of IκB, thereby liberating NF-κB dimers that translocate into the nucleus. NF-κB then binds to DNA at specific κB sites in the promoter regions that regulate target gene expression [28]. It has been reported that in hepatic cells, homocysteine-induced COX-2 expression is mediated via NF-κB activation [29]. However, whether NF-κB is involved in regulating the COX-2 expression in human monocytes in response to homocysteine needs to be elucidated. The results from the TF ELISA and ChIP assays of our present study demonstrated that the homocysteine stimulation increased the in vitro DNA binding activity and the in vivo COX-2-promoter binding of NF-κB in monocytes. This activation of NF-κB-DNA binding activity, induced by homocysteine, could be significantly inhibited by pretreating monocytes with NF-κB inhibitors PDTC or SN50, which could also inhibit the homocysteine-induced COX-2 expression in monocytes. Before stimulation with homocysteine, cells pretreated with FA significantly inhibited the homocysteine-induced NF-κB-DNA binding activity, as well as in vivo NF-κB-promoter binding in monocytes. These results suggest that the homocysteine and FA may share a common pathway, i.e., NF-κB, in mediating COX-2 expression in monocytes. Our results indicate that FA exerts an anti-inflammatory function on monocytes stimulated with homocysteine. In addition, these results also suggest that this FA inhibition of COX-2 expression stimulated with homocysteine may reflect the regulatory roles of FA in gene expressions in monocytes. The present data suggest that FA may serve anti-inflammatory and atheroprotective functions by inhibiting the pro-inflammatory gene expression in monocytes in response to homocysteine stimuli.

## Conclusion

In conclusion, FA inhibited the expression of COX-2 and production of PGE_2_ from homocysteine-induced monocytes. Stimulation of homocysteine in monocytes resulted in increased phosphorylation of ERK and JNK, and activation of NF-κB. FA inhibited homocysteine-induced inflammatory mediator expression by regulating the activation of these pathways.
